# Neurosurgical literature classification – Evaluation of three automated methods and time trend analysis of the literature

**DOI:** 10.1016/j.heliyon.2024.e26831

**Published:** 2024-02-21

**Authors:** Shayan Eftekhar, Behzad Eftekhar

**Affiliations:** a The University of Queensland, Brisbane, Australia; b Department of Neurosurgery, Nepean Clinical School, Sydney Medical School, Faculty of Medicine and Health, The University of Sydney, Sydney, Australia; c Department of Neurosurgery, Australian School of Advanced Medicine, Macquarie University, Sydney, Australia

**Keywords:** Neurosurgical literature, Text classification, Similarity-based methods, Human vs machine learning performance, Lbl2Vec

## Abstract

**Background:**

Automated supervised text classification methods require preclassified training data. Their application in scenarios that a large amount of preclassified data is not accessible is challenging. Neurosurgical literature classification into subspecialties is an example of this situation. We have introduced an automated similarity-based text classification method, evaluated it along with two other automated methods and applied the introduced method in neurosurgical literature classification.

**Methods:**

Performance of an introduced similarity-based text classification method along with two other automated methods (Lbl2Vec and keyword counting-based methods) was compared with performance of two senior neurosurgery registrars in classification of neurosurgical literature to 5 subspecialties. The Kappa-statistic measure of interrater agreement, overall marginal homogeneity using the Stuart-Maxwell test, marginal homogeneity relative to individual categories using McNemar tests and the sensitivity and specificity of each of the three methods were calculated.

The introduced method was used to classify 211617 neurosurgical publications indexed in Pubmed to different subspecialties based on keywords extracted from subspecialty sections of a neurosurgery textbook.

**Results:**

The introduced similarity-based method showed the highest agreement with the registrars (raw agreement and Kappa value) followed by the Lbl2Vec and the counting-based method. Classifications of the English neurosurgical publications indexed in Pubmed into categories of Oncology, Vascular, Spine and functional using the introduced similarity-based method were more reliable (closer to the registrars’ classifications) than Cranial trauma. The classifications and future forecast showed highest publications in Oncology, followed by Cranial trauma, Vascular, spine and functional neurosurgery.

**Conclusion:**

The classification of the English neurosurgical publications indexed in Pubmed to different subspecialties, using the introduced method, shows that Oncology and tumour has been the main battleground for the neurosurgeons over years and probably in the near future. The performance of the introduced classification method in comparison with the human performance shows its potential application in the situations that enough preclassified data are not accessible for automated text classification.

## 1 Introduction/Background

How can we find out what portion of the published literature by neurosurgeons are in different subspecialties and study its trend over years?

Specific keywords such as Medical Subject Headings (MeSH) and Boolean Operators are commonly used for specific search queries, however they have not been efficient in deeper analysis like thematic analysis or classification. Examples of thematic classification includes the above-mentioned query or classifying the surgical publications into basic science or clinical and either diagnostic, therapeutic or prognostic which traditionally requires going through the actual texts individually [[1], [2], [3]]. With a dramatic increase in the number of publications [4], traditional methods of examining papers from a large number of specific journals is no longer practical and efficient. Many automated methods have been suggested for text mining and classification with a growing number of publications about state-of-the-art machine learning algorithms being apparent [5,6], however, all supervised machine learning approaches require training by already classified large data sets which may be problematic in circumstances where enough training data may not be easily accessible. Our query is an example of these circumstances.

In this study using accessible text mining algorithms and codes, we have introduced a similarity-based approach for literature classification and compared its performance, along with the performance of two other automated classification methods, with human classification. Then the method has been utilized for the classification and time trend analysis of five subspecialties in neurosurgical English publications (indexed in Pubmed).

## 2 Methods

### 2.1 Data and extraction of keywords

Using E-utilities available through the National Library of Medicine website (https://dataguide.nlm.nih.gov/edirect/install.html), the PubMed database was searched using the keywords "Neurosurgery[AD] AND English[Language]" for publications indexed before July 30, 2022. 211617 records (Whole data) out of 242119 initially retrieved records, were included in the study due to the missing values from the remaining records. The title and abstract of each record were joined together and these joined texts were used for the classification process.

Two senior neurosurgery registrars (with more than 6 years neurosurgical experience) participated in classifying one hundred of the retrieved PubMed records which were randomly selected (Test data) to 6 categories of Vascular, Spine, Oncology, Functional, Cranial Trauma and ETC, independently.

**Fig. 1:**
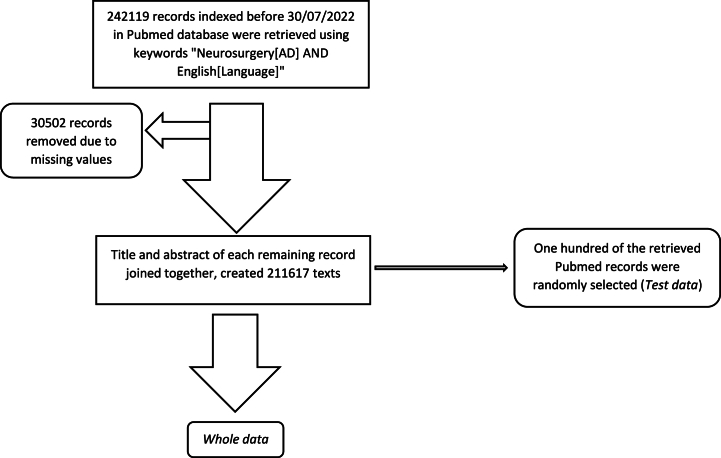
Flowchart for preparation of the data.

**Fig. 2:**
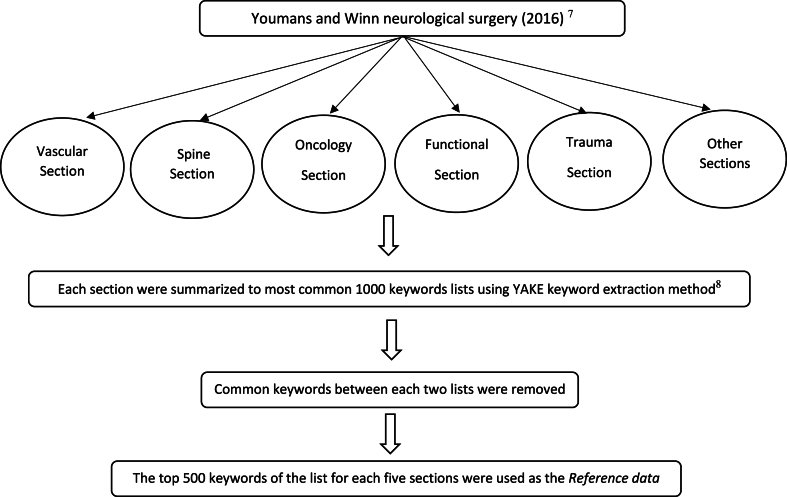
Flowchart for preparation of the keywords.

Texts of the five sections of Vascular, Spine, Oncology, Functional, Cranial Trauma as well as all other sections from Youmans and Winn neurological surgery [7] were summarized into the 1000 most common keywords lists using YAKE, which is an unsupervised learning keyword extraction method [8]. The keywords common between the lists were removed using Python scripts and the lists were reviewed by the authors. In order to reduce the bias due to the number of keywords, only the top 500 keywords of the list for each five sections were used as the Reference data. Fig. 1, Fig. 2 show the flowchart for preparation of the data and keywords.

### 2.2 Validation study

Part I: Classification of the Test data by Senior registrars.

Two senior neurosurgery registrars (with more than 6 years neurosurgical experience) participated in classifying one hundred of the retrieved PubMed records which were randomly selected (Test data) into 6 categories of Vascular, Spine, Oncology, Functional, Cranial Trauma and ETC, independently. No definition of the categories was provided to the registrars.

Part II: Classification of the Test data by simply counting the number of the keywords (Keyword Counting-based method)

Using Python scripts, the occurrence of the keywords from each of the 6 categories of Reference data were counted in each record of the Test data. Then for each record, the numbers of occurrence for the categories were compared using a T-Test statistical method. If there was a statistically significant difference (p < 0.05), then the record was allocated to the section with the highest number, otherwise the record was allocated to the ETC group.

Part III – Classifying the Test data by utilizing the plain Lbl2Vec algorithm using Doc2Vec (Lbl2Vec method)

Lbl2Vec is an Embedding-Based Approach for Unsupervised Document Retrieval on Predefined Topics [9,10]. We used the online codes developed by Tim Schopf (available from https://github.com/sebischair/Lbl2Vec and https://towardsdatascience.com/unsupervised-text-classification-with-lbl2vec-6c5e040354de) and tailored it to our study. The texts of each of the five sections of Vascular, Spine, Oncology, Functional, Cranial Trauma as well as all other sections from Youmans and Winn neurological surgery [7] were split into parts of 500 words after the removal of the stopwords and used for the training of a Lbl2Vec model. The Reference data was used as the “keywords_list”. After the model was trained, it was used to predict the categories of the Test data.

Part IV – Classifying the Test data by calculating the similarity between each record and the keywords of the Reference data (Similarity-based method)

The keywords for each section of the Reference data were joined together, building a text for each section made of keywords. The Soft Cosine Similarity index of the six sections of the Reference data with each record (title + abstract) of the Test data were calculated using available GENSIM open-source Python library and word2vec-google-news-300 model [[11], [12], [13]]. For each record of the Test data, the calculated similarity indices to each of the five sections of the Reference data were compared using a T-Test statistical method. If there were a statistically significant difference (p < 0.05), then the record was allocated to the section with highest similarity to the record (vascular, spine, trauma, oncology and functional), otherwise the record was allocated to the ETC group.

### 2.3 Comparison of different classifying methods

Using registrars’ classifications for cases where both agreed as the reference, for each category, the sensitivity (true positive/(true positive + false negative)) and specificity (true negative)/(true negative + false positive)) of each of the three methods was calculated using Diagtest module of STATA. The Kappa-statistic measure [14] of interrater agreement between registrars and three other methods, overall marginal homogeneity using the Stuart-Maxwell test and marginal homogeneity relative to individual categories using McNemar tests (a non-parametric statistical test that checks the marginal homogeneity of two dichotomous variables) were calculated using STATA MP 17 (StataCorp. 2021. *Stata Statistical Software: Release 17*. College Station, TX: StataCorp LLC).

### 2.4 Classification of the whole data

Considering the results of the Test data classification, the similarity-based method was used for the classification of the whole data. The frequencies of each category per year were plotted and extrapolated using Microsoft Excel (Microsoft Corp.) functions.

## 3 Results

The registrars classified 79 out of 100 records similarly (Kappa value of 0.7285 (p < 0.001)). Considering 21% disagreement between the registrars, classification results of those 79 records with similar classifications by the registrars were used as a reference standard to measure and compare the performance of the three other semi-automated classification methods used in this study.

The sensitivity and specificity of each of the three methods for each of the 6 categories (Vascular, Spine, Oncology, Functional, Cranial Trauma and ETC) were calculated separately (Table 1)

**Table 1:** The sensitivity and specificity of each of the three methods for each of the 6 classification categories.

	Similarity Sensitivity	Similarity Specificity	Lbl2Vec Sensitivity	Lbl2Vec Specificity	Keyword Sensitivity	Keyword Specificity
ETC	44.44%	98.08%	40.74%	94.23%	40.74%	82.69%
Vascular	100.00%	95.71%	88.89%	97.14%	55.56%	78.57%
Spine	86.67%	98.44%	60.00%	98.44%	46.67%	93.75%
Oncology	95.00%	93.22%	85.00%	94.92%	45.00%	96.61%
Functional	100.00%	97.37%	100.00%	96.05%	100.00%	93.42%
Cranial Trauma	80.00%	89.19%	80.00%	89.19%	40.00%	90.54%

The Kappa statistics were calculated to assess the level of agreement between reference standard and classifications using each of three above-mentioned methods.

Table 2 shows the results. The p-value of the Kappa statistics shows that classifiers agreement was more than chance alone would predict. As we can assume that all types of disagreements are equally "serious", the magnitude of Kappa can be interpreted as a form of intraclass correlation. The similarity-based method showed the highest agreement with the registrars (raw agreement and Kappa value) followed by the Lbl2Vec and the counting-based method.

**Table 2:** Kappa statistics measuring agreements between different groups – Classification results of those 79 records with similar classifications by the registrars were used as a reference standard (*the reference*) to measure and compare the performance of the three other semi-automated classification methods used in this study (see the text).

Groups	Number of Records	Agreement	Expected Agreement	Kappa	Std. err.	Z	Prob > Z
Two Senior Registrars classifications	100	79.00%	22.65%	0.7285	0.0501	14.55	0.0000
Classifications by Similarity-based method and *the reference*	79	75.95%	19.29%	0.7020	0.0526	13.35	0.0000
Classifications by Lbl2Vec method and *the reference*	79	67.09%	18.12%	0.5980	0.0503	11.88	0.0000
Classifications by Keyword counting-based method and *the reference*	79	46.84%	18.81%	0.3452	0.0513	6.73	0.0000

Table 3 shows the overall marginal homogeneity using the Stuart-Maxwell test. The test first tabulates the data in a 6 × 6 table and then performs [15] a test for table symmetry and the Stuart–Maxwell [16,17] test for marginal homogeneity. Both the symmetry test and the marginal homogeneity test are significant, thus indicating a significant difference between one or more of the classifications by the similarity-based method (SMB) and the reference standard.

**Table 3:** Overall marginal homogeneity using the Stuart-Maxwell test (SMB – similarity based method, please see the text, df – degree of freedom). Rows are the results of classifications by SMB and columns are results of classifications by the registrars. (e.g. from 79 records SMB classified 13 under ETC group and the registrars classified 27 under ETC group).

	reference standard (columns)						
SMB (rows)	ETC	Vascular	Spine	Oncology	Functional	Cranial Trauma	Total
ETC	12	0	1	0	0	0	13
Vascular	1	9	1	1	0	0	12
Spine	1	0	13	0	0	0	14
Oncology	3	0	0	19	0	1	23
Functional	2	0	0	0	3	0	5
Cranial Trauma	8	0	0	0	0	4	12
							
Total	27	9	15	20	3	5	79
		chi2	df	Prob > chi2			
Symmetry (asymptotic)		17	8	0.0301			
Marginal homogeneity (Stuart–Maxwell)		15.5	5	0.0084			

Table 4 shows the results of marginal homogeneity relative to individual categories using McNemar tests. There are statistically significant differences between the reference standard and the Similarity-based classifications in ETC and “Cranial Trauma” categories, making the performance of the Similarity-based method less reliable in these two categories.

**Table 4:** The results of marginal homogeneity relative to individual categories using McNemar tests.

Individual category	McNemar's chi2	Prob > chi2	Exact McNemar significance probability
Vascular	3.00	0.0833	0.2500
Spine	0.33	0.5637	1.0000
Oncology	1.80	0.1797	0.3750
Functional	2.00	0.1573	0.5000
Cranial trauma	5.44	0.0196	0.0391
ETC	12.25	0.0005	0.0005

**Fig. 3:**
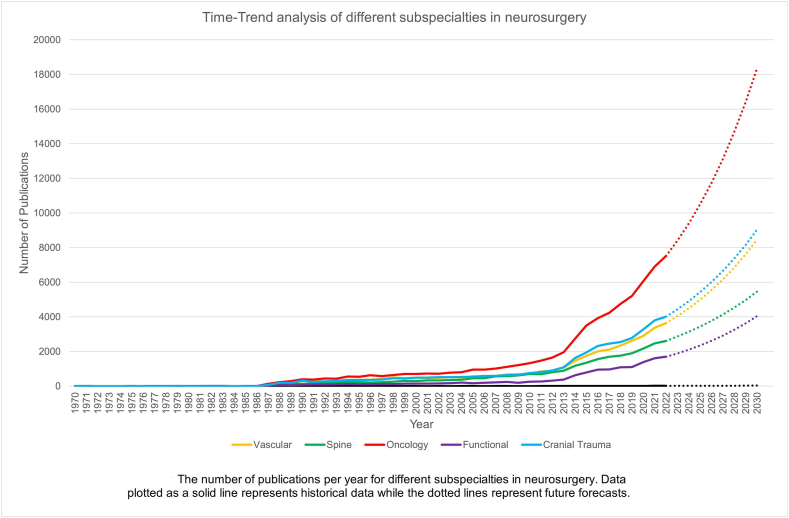
The number of publications per year for different subspecialties in Neurosurgery. Data plotted as a solid line represents historical data while the dotted lines represent future forecasts.

Fig. 3 shows the Time-Trend analysis with number of publications per year for different subspecialties in neurosurgery as well as the future forecasts.

## 4 Discussion

The automated text classification methods can be divided into two major categories: supervised and unsupervised. While both supervised and unsupervised approaches to literature classification are based on a statistical analysis of the words used [18], their methods and interpretation differ fundamentally. Supervised classification models are trained on a ground truth, which could be an existing classification carried out by subject-matter experts. The supervised classification learns parameters based on the distribution of words in the ground truth that is used later to classify unclassified texts. Hence, accuracy has the natural interpretation of how well the classifier replicates the ground truth. On the other hand, unsupervised classification, does not require any pre-existing ground truth. Instead, unsupervised classification depends on model parameters given [19,20].

We focused predominantly on unsupervised or semi-supervised text classification techniques that do not require having a large number of annotated data for text classification tasks. Our preliminary experiments with other unsupervised or semi-supervised methods did not provide the desired outcome, hence we introduced a similarity-based text classification method based on available algorithms and codes.

The closest unsupervised method to our approach is work of Haj-Yahia et al. [21] using keyword enrichment and subsequent unsupervised classification based on latent semantic analysis and cosine similarities. Both approaches are based on the calculation of text similarity between two sets of words: One containing the relevant words in the document and another containing keywords related to the target categories (Reference data in our study). Our approach was simpler and differed slightly from their work, as we did not have to go through their four keyword enrichment steps and we removed the common keywords between categories of Reference data, so only one category contained a given keyword. We also limited the number of keywords to the top 500 keywords of the list for each of the five categories of the Reference data to reduce the risk of bias towards any one of the categories due to the number of the keywords.

In our study we compared three automated methods of text classifications with the classifications done by senior neurosurgery registrars as the Reference data. The similarity-based approach showed highest agreement with the Reference data and was used to classify the neurosurgical publications indexed in PubMed from 1970 to 2021. Our classifications showed the dominance of Oncology related publications during all these years.

We considered the classifications done by the senior neurosurgery registrars as the ground truth. 21% disagreement between two registrars makes this assumption debatable and shows the inherent difficulty of classifications and overlap between neurosurgical subspecialty texts. The agreement between registrars’ classifications could improve with provision of feedbacks and more strict category definitions, however it was avoided on purpose as these disagreements reflect the true nature of the task and should be taken into consideration in evaluation of any classification methods.

Lbl2Vec approach has performed better in similar studies [9]. Splitting the texts of each section of a neurosurgical textbook to 500 words parts and using them as labelled training instances assumes that the split parts of each section classify as the same category as the whole section which may not be true and should be considered when measuring the performance of Lbl2Vec method. Having said that, the main challenge with our query and similar queries is lack of enough preclassified data that could be used for training a Domain-Specific Word Embedding Model. In fact, one of the advantages of the introduced method (similarity-based approach) is to perform categorization without using large amount of annotated data during training and therefore offer the potential to reduce annotation costs. The study shows the potential of the proposed method for similar tasks not only in English but also in non-English literature.

We used Yake keyword extraction and Soft Cosine Similarity index in our study as a proof of concept. There are other methods of keyword extraction and estimating the degree of similarity between texts that can be used for the same purpose. The advantages and disadvantages of these methods have been discussed in the literature [22,23].

In order to minimize the chance of bias, the number of words in each section of the reference data should be preferably the same. We summarized the texts to 500 keywords. Summarization of the text using keyword extraction methods may not be necessary in other scenarios which the known texts are already short with similar lengths.

Considering lacking a definitive criterion variable or "gold standard" in our literature classification and using nominal ratings, Kappa statistics were more suitable for our performance assessments. Based on the statistical tests, it is reasonable to consider that our classifications in categories of Oncology, Vascular, Spine and functional more reliable (closer to the registrars’ classification) than Cranial trauma. We could not find similar study to compare the results of our neurosurgical literature classification with.

Taking into considerations the above-mentioned points, the results of the study in terms of the past and future trends of the publications seem to be a true reflection of the real world with dominance of oncology and tumour as the main battleground for the neurosurgeons over years and probably in the near future. The results of the study agree with published data regarding the upward global trends of brain and central nervous system (CNS) cancer. Fan Y. et al. [24] reported in 2019, a significant increase (94.35%) from the period between 1990 and 2019. The upward trend not only can be explained with increased incidence of CNS cancers but also with ongoing advances in technological aids to diagnosis, surgical management and adjuvant therapies.

The results of our neurosurgical literature classification and the methodology of our study can be considered as the novel aspects of our study. Further evaluation of the introduced method in other contexts, particularly circumstances where enough training data may not be easily accessible, is suggested.

## 5 Limitations

1 - We used the texts of each of the five sections (Vascular, Spine, Oncology, Functional and Cranial Trauma) of a popular textbook of Neurosurgery and split them into parts of 500 words after the removal of the stopwords and used them for the training of a Lbl2Vec model. While it is expected that the majority of the texts in each section be related to the topics (Vascular, Spine, Oncology, Functional or Cranial Trauma), it can be argued that there is possibility that parts of each section (with 500 words length) may not fully relate to their section topic when considered out of the context or could be labelled for more than one topic. For example, in Oncology section of the textbook, there might be a part discussing vascularity of the brain tumours. This part is labelled as Oncology while out of context could be labelled Vascular as well. This limitation underestimates the Lbl2Vec classification method performance in our assessment.
2 - We have not explored Bidirectional Encoder Representations from Transformers (BERT) domain-specific pretrained models (e.g. BioBERT [25] and PubMedBERT [26]). BERT is based on the Transformer encoder and has been trained on the masked language model and next sentence prediction tasks. However, for text classifications it can be used for capturing the semantics of the text and use this embedding as the input of a classifier. Recent studies have been promising showing that domain-specific pretraining can serve as a solid foundation for a wide range of biomedical natural language processing tasks [26]. It is increasingly common to use pre-trained models that provide contextualized embedding in various semantic analysis/matching/comparison tasks. However, the matching conducted in the present study is on the level of word sets, and probably would not benefit much from the use of such contextualized embeddings.
3 - We used PubMed as it has made its data available for similar studies. PubMed and the search query "Neurosurgery[AD] AND English[Language]" not cover all neurosurgical publications.
4 - When interpreting the trend of the publications, it should be taken into consideration that in the Reference data, the spinal trauma has been covered in the spine section and there are specialties other than neurosurgery who perform research in the studied fields such as Orthopaedic spine surgeons in the field of spine or neurologists in functional and vascular. In other words, our study and final graph may not reflect the reality about the trend of publications in the field of spinal surgery.

## 6 Conclusion

In this study the authors discussed the challenges of neurosurgical thematic text classifications, introduced a semi-supervised similarity-based text classification method and evaluated it along with two other automated text classification methods against human performance. The classification of the English neurosurgical publications indexed in PubMed to different subspecialties, using the introduced method, shows the dominance of oncology and tumour as the main battleground for the neurosurgeons over years and probably in the near future. The performance of the introduced classification method in comparison with the human performance shows its potential application in the situations that enough labelled instances are not accessible for training of Domain-Specific Word Embedding Models.

## Data availability statement

Data associated with this study has not been deposited into a publicly available repository. Data will be made available on request.

## Additional information

No additional information is available for this paper.

## Disclosure of Funding

None.

## CRediT authorship contribution statement

**Shayan Eftekhar:** Writing – review & editing, Visualization, Formal analysis, Data curation. **Behzad Eftekhar:** Writing – review & editing, Writing – original draft, Supervision, Methodology, Formal analysis, Conceptualization.

## Declaration of competing interest

The authors declare that they have no known competing financial interests or personal relationships that could have appeared to influence the work reported in this paper.

## References

[1] Comish P.B., Madni T.D., Nakonezny P.A. (2021). An analysis of surgical literature trends over four decades. Am. J. Surg..

[2] Baek S., Yoon D.Y., Min K.J., Lim K.J., Seo Y.L., Yun E.J. (2014). Characteristics and trends of research on positron emission tomography: a bibliometric analysis, 2002–2012. Ann. Nucl. Med..

[3] Shamim M.S., Enam S.A., Kazim S.F. (2011). Neurosurgical research in Pakistan: trends of publication and quality of evidence. Clin. Neurol. Neurosurg..

[4] Kyvik S., Aksnes D.W. (2015). Explaining the increase in publication productivity among academic staff: a generational perspective. Stud. High Educ..

[5] Thangaraj M., Sivakami M. (2018). Text classification techniques: a literature review. Interdiscipl. J. Inf. Knowl. Manag..

[6] Li R. (2022).

[7] Winn H.R. (2016).

[8] Campos R., Mangaravite V., Pasquali A., Jorge A., Nunes C., Jatowt A. (2020). Yake! Keyword extraction from single documents using multiple local features. Inf. Sci..

[9] Schopf T., Braun D., Matthes F. (2022).

[10] Schopf T., Braun D., Matthes F. (2022).

[11] Sidorov G., Gelbukh A., Gómez-Adorno H., Pinto D. (2014). Soft similarity and soft cosine measure: similarity of features in vector space model. Comput. Sist..

[12] Novotný V. (2018).

[13] Mikolov T., Chen K., Corrado G., Dean J. (2013).

[14] McHugh M.L. (2012). Interrater reliability: the kappa statistic. Biochem. Med..

[15] Bowker A.H. (1948). A test for symmetry in contingency tables. J. Am. Stat. Assoc..

[16] Maxwell A.E. (1970). Comparing the classification of subjects by two independent judges. Br. J. Psychiatr..

[17] Stuart A. (1955). A test for homogeneity of the marginal distributions in a two-way classification. Biometrika.

[18] Lee C.-H., Yang H.-C. (2009). Construction of supervised and unsupervised learning systems for multilingual text categorization. Expert Syst. Appl..

[19] Goh Y.C., Cai X.Q., Theseira W., Ko G., Khor K.A. (2020). Evaluating human versus machine learning performance in classifying research abstracts. Scientometrics.

[20] Minaee S., Kalchbrenner N., Cambria E., Nikzad N., Chenaghlu M., Gao J. (2021). Deep learning--based text classification: a comprehensive review. ACM Comput. Surv..

[21] Haj-Yahia Z., Sieg A., Deleris L.A. (2019).

[22] Firoozeh N., Nazarenko A., Alizon F., Daille B. (2020). Keyword extraction: Issues and methods. Nat. Lang. Eng..

[23] Prakoso D.W., Abdi A., Amrit C. (2021). Short text similarity measurement methods: a review. Soft Comput..

[24] Fan Y., Zhang X., Gao C. (2022). Burden and trends of brain and central nervous system cancer from 1990 to 2019 at the global, regional, and country levels. Arch. Publ. Health.

[25] Lee J., Yoon W., Kim S. (2020). BioBERT: a pre-trained biomedical language representation model for biomedical text mining. Bioinformatics.

[26] Gu Y., Tinn R., Cheng H. (2021). Domain-specific language model pretraining for biomedical natural language processing. ACM Transactions on Computing for Healthcare (HEALTH).

